# Specific Medication

**Published:** 1880-08

**Authors:** Dudley M. Culver

**Affiliations:** Whitesville, Ind.


ARTICLE IV.

### SPECIFIC MEDICATION.

BY DUDLEY M. CULVER, M. D., WHITESVILLE, IND.

I am sorry to see members of as high a calling as the medical profession, allowing themselves to be led off by pharmaceutical laboratories into what is termed "Specific Medication," and it is upon this subject that I desire to be chiefly heard for the present.

What does Specific Medication mean? It means "quackery" and nothing more. A man away over in California, signs his name M. D. He has found what he *claims* to be a plant indigenous to that state, and he calls it "Cascara Sagrada" "*Raumus Purshiana.*" He holds it up by the tail, and employs a pharmaceutical establishment to manufacture and advertise it for him. They come out in bold wrappers: "Cascara Sagrada," a sure *cure* for Habitual Constipation, Dyspepsia, Paralysis, Neuralgia of the stomach, &c., &c.

Now, brethren, I ask in the name of reason, if this is not leaving principle altogether, and following up quackery and empiricism? Why not just as well prescribe Bull's Baby Syrup, Smith's Tonic Syrup, and allied preparations? They are put up as neatly, have no more printed matter around them, and prove just as efficacious. Don't we know we would be laughed at if we should be found prescribing patent medicines? Most certainly we would. Then I desire to impress the profession wlth the necessity of practicing medicine on a scientific basis. Use reason and not specific medicines. Here we have a case of Constipation; science teaches us to inquire into the cause; remove that first, and afterward the effects. Now, is your constipation habitual, or is it accidental? Is it due to deficient flow of bile, deficiency in intestinal juices, or is it due to paralysis of the nerves which supply the muscular coat of the intestines? Or is it due to impacted feces? All of these causes may produce constipation, and a man with a thimble full of brains would know that what would remove the constipation under one cause would not under the other, but on finding the *cause* each will require a different plan of treatment. Can we suppose, for instance, that Cascara Sagrada would remove impacted feces any better or as well as the finger, or blunt spoon? Answer we, "specfic." Then science interposes and demands that we seek for the cause, remove that, and afterwards should they exist, the effects of the cause.

What a pleasure to meet in consultation with Dr. A. He has a case of constipation on his hands. We ask him what his course of treatment has been. He tells us: Well, eh! it's constipation. I've been treating it with specific "Cascara Sagrada." Yes—well Dr. A., on what principle do you give "Cascara," what physiological effects do you expect from it? Ans.: Well, I expect it to remove constipation, that's all I can tell you; in fact, that's all I am taught about it. Now, don't you know it would be a pleasure to counsel with a man of this kind, but gentlemen of the medical profession, this is where Specific Medication is leading us to! Again, we have a medicine "Coto Bark," recommended for Diarrhœa, Dysentery, Gout and Rheumatism.

This beats all of them. We shall first examine it as a remedy for diarrhœa; now, we know that diarrhœa, like constipation, assumes different forms and is produced by as many different causes, and without consuming space to enumerate the different causes and forms of diarrhœa, we will be content with saying, how fallacious the idea of having a specific for diarrhœa. We have, for instance, as we all know, some forms of diarrhœa in which there are foul secretions and crude ingesta in the intestines, and that would result in danger and possibly death to our patient to use a remedy to *check* the bowels without first ridding them of these poisonous and irritating principles, then *curing*, actually *curing* rheumatism and gout, with a diarrhœa mixture. Pray, brethren of the "healing band," whither are we drifting? When we prescribe for any disease, we ought, if possible, to know first what we are prescribing for, and what we are prescribing. We should try to be able, as Paul says: "To give a reason for all things!" no satisfaction is felt when you prescribe for a case, if you don't know *why* you are prescribing, or what the physiological effect of the medicine will be on the system! Then let "Specific medicine" go by the board, for it must prove disastrous to true Medical Science should it obtain a foothold. But should we come and reason together, and exercise sound judgment, instead of Routine Skepticism success will crown our efforts. Now I am not attempting to detract from nor ridicule the manufacturing Pharmacists who prepare these medicines, for "Coto Bark" is a good remedy in some forms of Diarrhœa: and "Cascara Sagrada" *is* a good remedy in some cases of Constipation. But we dont want them as "specifics." We don't want printed labels on the bottle telling of a score of diseases for which it is good. But what we do want is the name of the medicine, dose, and its "*Physiological*" action. Nothing more. Nothing less. Then let the physician choose for himself in what diseases or peculiar conditions, he will apply it. "Coto Bark" is advertised as a Specific in Diarrhœa, Dysentery, Gout, Rheumatism, &c., and is therefore claiming or assuming too much for it. It is too much on the "Patent medicine" plan and savors of quackery, &c. We don't want any of it. We like "Coto Bark" so far as we have tested it, and we think that we are safe in saying that, Parke Davis & Co., manufacture a pure, and reliable article; but we don't want them to prescribe it; and put patent medicine wrappers around their bottles, we don't want any "Specific Medication." Then gentlemen come to the front ranks, let us use common sense, come forward and express your opinions and let us sift the matter and see if "Specific medication" isn't worse than a farce and delusion. I submit the matter to the candid reader, in the hope that we may" do the greatest good to the greatest number. I would like to say more but limited space forbids.

